# Patients' Perspectives on Participation in an Effectiveness Study on Footwear Modification for the First Metatarsophalangeal Joint Osteoarthritis: A Qualitative Study

**DOI:** 10.1002/jfa2.70050

**Published:** 2025-04-23

**Authors:** Carla Braam, Sabine Kloprogge, Dieuwke Schiphof, Joyce B. J. van Meurs, Sita M. A. Bierma‐Zeinstra, Marienke van Middelkoop

**Affiliations:** ^1^ Department of General Practice Erasmus MC University of Medical Centre Rotterdam the Netherlands; ^2^ Department of Internal Medicine Erasmus MC University of Medical Centre Rotterdam the Netherlands

**Keywords:** first metatarsophalangeal joint osteoarthritis, footwear modifications, general practice, patients' perspectives, primary healthcare, qualitative research

## Abstract

**Background:**

The effectiveness of footwear modifications for the first metatarsophalangeal (MTP) joint osteoarthritis (OA) compared to usual general practitioner (GP) care has never been studied. Understanding patients' perspectives is essential for assessing the feasibility of a randomized controlled trial (RCT) on this topic. Our objective is to explore experiences, expectations, beliefs, and opinions of patients with the first MTP joint OA regarding symptoms and limitations, healthcare management, footwear intervention, and research participation for designing a successful future effectiveness trial.

**Method:**

A qualitative research design was embedded within a feasibility pilot study for the recruitment of participants diagnosed with the first MTP joint OA. Qualitative data from semistructured interviews were categorized analyzed.

**Results:**

All participants (*n* = 10) experienced limitations on the activity and participation level due to pain symptoms in the first MTP joint. Patients experienced varying approaches and treatment outcomes in primary and secondary healthcare, leading to both positive and negative perspectives. Most participants highlighted the importance of the cosmetic appearance of modified footwear, indicating that this is crucial for compliance with the intervention. Participants showed willingness to participate in an RCT, with strong preference for randomization into an intervention group with a modified footwear alongside usual GP care rather than GP care alone.

**Conclusion:**

Our study identified key considerations for designing a successful future trial, including recruiting incident cases, offering the deferred footwear intervention to the control group, providing clear information during recruitment and randomization phase, and the significance of the cosmetic appearance of modified footwear for patients with the first MTP joint OA.

## 1 Introduction

Foot pain is very common in the general population. Almost one in four adults of middle and old age suffers from foot pain on a daily basis [1]. Foot osteoarthritis (OA) is one of the conditions that can cause foot pain. First metatarsophalangeal (MTP) joint OA is the most common form of foot OA and affects 7.8% of people aged 50 years and over [2]. People with a symptomatic first MTP joint OA typically present with localized pain, stiffness, and an enlarged joint [3, 4, 5], which leads to substantial problems in performing functional weight bearing activities and is associated with a high impact on foot‐specific health‐related quality of life [3]. Therefore, effective conservative treatment strategies that aim to improve pain and symptoms as well as health‐related quality of life are needed [6].

Conservative treatments for an early‐stage first MTP joint OA typically include footwear modifications and orthotics [6, 7], to relieve pain by modifying load distribution of the foot and reducing load through the first MTP joint when walking [8]. Modifications of ready‐made shoes can consist of stiff insoles, widening shoe parts, shaft stiffening, and/or rocker soles to restrict toe plantarflexion to ensure proper offloading of the forefoot and reduce peak pressure beneath the first MTP joint [9, 10, 11, 12]. Footwear interventions seem to be associated with improvements in pain and function of the first MTP joint [13].

A limited number of studies have so far investigated the effectiveness of interventions for the first MTP joint OA [14]. Most studies have been conducted in secondary healthcare settings, with a primary focus on invasive surgical interventions [15]. With the exception of three Australian studies that examined the effectivity of various footwear modifications [16, 17, 18], conservative intervention trials are lacking [14, 19]. This is also confirmed in a recently published Cochrane review by Munteanu et al. (2024), who concluded that there is limited evidence regarding the benefits of conservative treatments for the first MTP joint OA [19]. Therefore, there is an urgency to study the cost‐effectiveness of footwear interventions in primary healthcare, in particular, the efficacy of footwear modifications of ready‐made shoes, which has never been studied before.

However, trials are challenging in primary healthcare settings, and success is dependent on the study design and on the willingness and compliance of participants with the intervention [20, 21]. To be able to conduct a successful randomized controlled trial (RCT) on modifications of ready‐made shoes, insight in patients' willingness and compliance with the intervention is particularly useful. Consumer input provides critical information to inform the study design [22] and can improve recruitment in clinical trials, especially when consumers with lived experience of the health condition are involved [23]. The perspectives of patients with the first MTP joint OA on trial participation have not yet been considered. Therefore, the objective was to gain a deeper understanding of patients' experienced symptoms and limitations, their healthcare pathway, their expectations of and compliance with the intervention, and their willingness and opinion regarding participation in a trial within primary healthcare to optimize the study design for a successful future RCT.

## 2 Methods

### 2.1 Study Design

A qualitative study design with individual semistructured interviews was used to explore patients' perspectives on four topics: (i) experienced symptoms and limitations when diagnosed with the first MTP joint OA; (ii) primary and secondary healthcare management; (iii) usability and acceptability of the footwear intervention; and (iv) patients' perspectives on research participation.

Considering the limited knowledge available on the four topics in the first MTP joint OA patients, deductive content analysis was performed on these predetermined topics (categories). Additionally, inductive content analysis was performed when new subcategories emerged in the interviews [24]. We followed the Standards for Reporting Qualitative Research (SRQR) (see Supporting Information S1: Appendix A). The study was conducted in accordance with the Declaration of Helsinki, and the ethical approval was obtained from the Medical Ethical Committee of Erasmus Medical Center (MEC‐2022‐0723).

### 2.2 Participant Selection and Recruitment

This qualitative study was nested in a quantitative pilot study designed to assess the feasibility of recruiting participants diagnosed with an early first MTP joint OA (< 2 years) and aged 45 and over. Participants in the region of Rotterdam, The Netherlands were recruited for this pilot study between April and July 2023 via an academic network of general practitioners (GP), social media platforms, newspaper advertorials, and local newspaper advertisements. From 54 screened volunteers from the quantitative pilot study, 17 potential participants (included or excluded for several reasons in the pilot study) were selected in June and July 2023 for the qualitative study. Participants for the current study had to be aged over 45 and a confirmed diagnosis of MTP joint OA. Purposive sampling was utilized to select participants based on the distribution by age, sex, symptom duration (< 2 years and ≤ 2 years), pain symptoms present on most days of last month, and previous experiences with primary and/or secondary care in relation to the first MTP joint OA. Potential eligible participants were emailed about the interview procedure and topics and asked about their willingness to participate.

### 2.3 Data Collection

Ten participants agreed to participate and individual semistructured interviews were conducted between July 2023 and September 2023 using an interview guide (see Supporting Information S1: Appendix B). The interview guide was developed with topics and questions derived from insights from our pilot study. Prior to data collection, the concept interview guide was tested in a practice session with a patient with the first MTP joint OA who was not included in the study, under supervision of a member of the authorship team (DS) who provided feedback. Consequently, minor adjustments were made to the interview guide, that is, rewording questions to increase understandability. The interview duration ranged from 27 to 62 min. All interviews were conducted by CB via an online video conferencing portal (Microsoft Teams). All participants gave verbal informed consent prior to the start of the interview and were informed that they could stop the interview at any time. Each informed consent and interview was audio‐recorded and video‐recorded for transcription purposes. Pretranscripts, automatically generated transcripts by Microsoft Teams, were directly provided during all interviews. Confidentiality was maintained using restricted secure access to the data. The identity of the participants in the pretranscripts was replaced with a study number and each pretranscript was checked and corrected by a member of the research team and stored as anonymized transcripts.

Prior to the interviews, general characteristics of the interviewed participants were derived verbally, that is, age, sex, weight, length, working status, and highest completed education level. After each interview, participants were asked if they wished to receive the transcript.

### 2.4 Data Analysis

For data analysis, elements of the methodology of the grounded theory approach were applied, specifically the method of open, axial, and selective coding [25]. First, within the four predescribed categories, performed by deductive content analysis, subcategories were derived using inductive content analysis. Open coding was applied on two interviews by labeling quotes to the subcategories by CB in accordance with three members of the authorship team (SK, DS, and MvM). This resulted in redefinement of some subcategories. Subsequently, axial coding was applied in four interviews independently by CB and SK. Differences in coding were discussed and redefined again if needed until consensus was reached with a third researcher (DS). Finally, selective coding was conducted on six interviews, including those in which open coding was applied. Data analysis process was facilitated using the software program MAXQDA version 2018.

In addition, we described general characteristics of the interviewed participants (i.e., age, sex, symptom duration (< 2 years and ≥ 2 years), experiences with primary and/or secondary healthcare, and socioeconomic aspects).

## 3 Results

**FIGURE 1:**
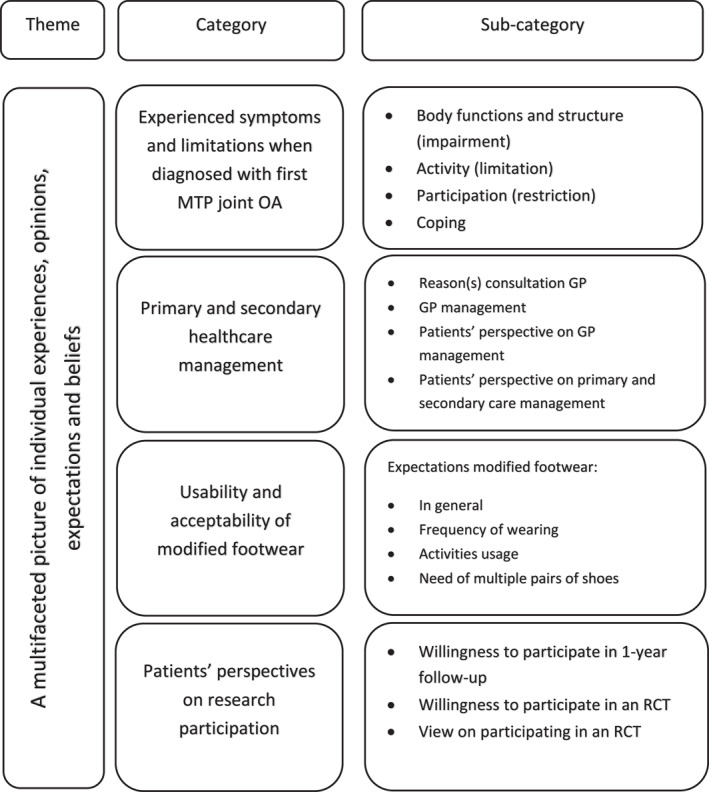
Overview of the theme, categories, and subcategories describing patients' perspectives.

Ten participants were interviewed. The participants' characteristics are presented in Table 1. The analysis resulted in descriptive subcategories derived from the four predetermined categories: participants' perspectives on (i) experienced symptoms and limitations when diagnosed with the first MTP joint OA; (ii) primary and secondary healthcare management; (iii) usability and acceptability of the modified footwear; and (iv) research participation. From those categories and the derived subcategories, one theme of meaning was formulated: “A multifaceted picture of individual experiences, opinions, expectations, and beliefs” (Figure 1). The theme illustrates how the participants expressed a broad range of topics in their perspectives on the first MTP joint OA and are in further detail for each category described below.

**TABLE 1:** Participant characteristics.

Participant ID	Age	Sex	Symptom duration	Experiences with primary and secondary care	Working status	Highest completed education level
1	62	M	A	Primary	Paid employment	High
2	45	F	A	Primary	Paid employment	Secondary
3	73	M	B	Primary and secondary	Retirement	Academic
4	60	F	B	Primary and secondary	Paid employment	Academic
5	55	F	B	Primary and secondary	Paid employment	Intermediate
6	50	F	A	Primary	Freelancer	High
7	51	F	B	Primary and secondary	Paid employment + freelancer	High
8	60	F	A	Primary and secondary	Nonworking	High
9	46	F	B	Primary and secondary	Paid employment	Intermediate
10	62	F	A	Primary	Incapacitated/disabled	Primary

*Note:* M male, F female, A < 2 years, and B ≥ 2 years.

### 3.1 Experienced Symptoms and Limitations When Diagnosed With the First MTP Joint OA

All participants reported experiencing a stabbing, nagging, and/or continuous pain when loading the foot and also at rest clearly illustrated by participant‐8 *“The main symptom is, yes, pain while walking, of those horrible stitches now and then. And yes, very severe pain in the toe, even at rest”.* From a mild to severe degree, all participants experienced limitations while walking. Each participant described to deal with this walking limitation differently. The following quotes illustrate this clearly: participant‐6 *“Yes, it is especially during walking when you notice it of course..… so it's not that I stop walking, so I accept that moment”.* Participant‐10 *“I have a small dog….. And normally, I very much like to do also walking and that limits me very much at the moment. I hate that. Then I go to the park with him. The end of the street I think, oh no. Then it becomes a small round for him”.*

Most participants experienced limitations in participating in work, sports, or hobby activities. Participant‐7 explained: “*I used to do fitness a lot too.…. But for everything you actually use your feet. So anyway, exercising has just become an obstacle*”.

Participant‐4 illustrated how she deals with the symptoms that she experiences from the first MTP joint OA during her work: “*But suppose I have a meeting and I want to have a neat shoe on then. Then I must consider it.… I put on those neat shoes in the toilet, so to speak, just before I walk into a meeting*”.

### 3.2 Primary and Secondary Healthcare Management With the First MTP Joint OA

All participants indicated that they consulted a GP for the first MTP joint pain. Most participants did not seek immediate medical GP attention upon the onset of symptoms. Participant‐6 said: *“Yes, after 6 months of experiencing symptoms, I went to see my GP”.*

There seemed to be a noticeable variation in the GP management as reported by participants. Six out of 10 participants indicated that the GP chose a “watch and wait” policy first. Four of these participants felt that there was need for a more proactive approach from the GP illustrated by participant‐9: *“Well, she could have already suggested earlier to take an X‐ray or a consult with the orthopedic surgeon, or well, something like that”.*

Two participants received painkillers and one had an intra‐articular injection from their GP. GPs referred three participants to a podiatrist and five to an orthopedic specialist at a subsequent consultation. Five participants viewed GP management positively, with most experiencing it as shared decision‐making, illustrated by participant‐1: *“Yes, it's fine. I have a good relationship with the GP. Everything is open for discussion and choices can be made”.*

Participants had varied experiences and opinions on primary and secondary healthcare interventions. Primary care, mainly provided by podiatrists, included in all cases provision of orthotics. Although participants were generally positive about the care provided, they were negative about its effectiveness on pain. One of the answers to the question “What did you think of the podiatrist's treatment” is illustrated by participant‐8: *“Yes, okay. But it did not satisfy. I remained in pain. I walk with it and occasionally not with those orthotics”.*

In secondary healthcare, consultation with the orthopedic specialist was perceived as being clear and enlightening. Participant‐4 expressed her opinion about her consult with an orthopedic specialist: *“The orthopedic specialist was quite brief and to the point, I must say….. And I found that to be useful information”.* All participants with the long‐term symptom duration (≥ 2 years) consulted the orthopedic specialist at least once.

### 3.3 Usability and Acceptability of the Footwear Intervention

All participants agreed that cosmetic appearance is relevant when wearing modified footwear. Orthotics did not provide any resistance from the participants. However, it was indicated that modifications to the external appearance of the ready‐made shoes should be subtle. When modifications were overly conspicuous, some participants stated that they would only utilize the footwear for specific activities. Participant‐4 expressed her opinion about shoe adjustments of custom‐made footwear: *“I would really appreciate it if it could be done with my own shoes, but it depends on how visible it is”.*

All participants expected that they would like to wear modified footwear daily for outdoor activities. Participant‐1: *“A lot for outside. That's yes, just when I walk outside. And to work, and yes, when I go shopping, go to town, things like that”*. Furthermore, all participants expected to wear modified footwear in every season of the year. This aligned with participants need for multiple modified shoes for both summer and winter. Participant‐5: “*But yes, I think, initially it would be nice to have a summer pair, a winter pair, and a pair for formal occasions.”*

### 3.4 Patients' Perspectives on Research Participation

Most (7 out of 10) participants indicated that they were willing to participate in an RCT. Two of these participants explicitly indicated their preference for randomization into the intervention group illustrated by Participant‐6: *“Yes, I think I would still participate, but I might be quite disappointed if I ended up in the control group, so to speak”.* In contrast, one participant considered a benefit of randomization in the control group, participant‐5: *“Look, now I can decide for myself what shoes to wear, and in that group, of course, I can't. Look, and imagine if you have your own shoes and a painkiller, and it works too. Yes, then I would also be happy.”*

Two other participants with short‐term symptoms clearly differed in their opinion about their willingness to participate in an RCT. These contrary beliefs are illustrated by the following quotes: participant‐8 preferred to choose her own care pathway, *“I understand the need for it. It's just random where you come in……But myself, I would struggle with that right now, because I really just, I want to move forward still, yeah.”* Participant‐10 saw the benefits of participation to address pain: *“If something can be done about it, please. I would like to participate in the study, with the hope that I can get rid of the pain and start walking normally again.”*

Participants gave multiple considerations on participation in an RCT, including the study duration, incurring costs, degree of pain, and the consideration that usual GP care has already taken place. Three participants felt that they would not benefit from receiving usual GP care alone in the control group. Therefore, they indicated to be unwilling to participate in an RCT. Participant‐3: *“I would like to help improve science. But I must be seriously wiser myself. And if not, then I drop out, yes.”*

All participants indicated to be willing to participate in a 12 months follow‐up quantitative study with quarterly questionnaires. The majority was completely open to it, except for one participant: participant‐3: *“Then something must make me wiser. If that's not the case, and you guys still just want to send me quarterly surveys. Do me a favor… Then there should also be an intervention. Yes, yes.”*

## 4 Discussion

The results of this study indicate that patients with the first MTP joint OA primarily experienced limitations in walking and other activities due to pain. Patients' experiences with primary and secondary healthcare varied in approach and treatment outcomes. Almost all participants emphasized the importance of the appearance of custom‐made footwear for intervention adherence. Participants were willing to participate in an RCT but preferred randomization into an intervention group in addition to usual GP care compared to usual GP care alone or were opposed to that control intervention. We identified no discernible differences across the various participant characteristics in experiences, opinions, expectations, and beliefs within the subcategories.

Many challenges are faced when comparing a footwear intervention with usual GP care versus usual GP care alone in a randomized controlled trial. Based on the interviews with our study participants, it is advisable to consider several aspects before conducting such an RCT. People with the symptomatic first MTP joint OA experience more foot pain, have greater difficulty performing functional weight‐bearing activities, find it more difficult to obtain suitable footwear, and perceive their feet to be in a poorer state of health than those without this condition [3]. This aligns with findings in our study, where all participants reported difficulty performing a broad range of physical activities due to symptoms of the first MTP joint OA. Pain associated with joint discomfort is highly variable and evolves over time [26]. The joint pain of OA is typically described as being exacerbated by activity and relieved by rest [27]. As a result of the progressive nature of the disease, increasing pain is often a reason to seek medical care. Similarly, in our study, most participants indicated that increasing pain and joint discomfort were reasons for GP consultation and they did not consult their GP immediately upon experiencing initial symptoms of pain of the first MTP joint. It is not only patients presenting with the initial symptoms of the first MTP joint OA who may defer consulting their GP. GPs may also be disinclined to diagnose OA directly at first presentation and may be content to do so after a policy of “watch and wait,” which may allow symptoms to worsen in order to justify or support the diagnosis [28]. This may relate to the fact that many of our study participants indicated that their GP initially adopted a ‘watch and wait' policy. Another explanation for the ‘watch and wait' approach of the GP might be the current lack of (inter)national guidelines for the management of the first MTP joint OA [29]. Treatment options for the first MTP joint OA are diverse and range from conservative management to invasive surgical interventions [30]. The evidence supporting these treatments is limited, and their efficacy remains uncertain [14, 19]. Therefore, in the absence of a guideline for managing the first MTP joint OA, targeted care will be limited, leading GPs to adopt a “watch and wait” policy.

The cosmetic appearance of the footwear intervention was very important to participants and thus will affect its use. As better compliance is associated with cosmetic appearance of the footwear intervention, it is not only important for the patients but also for the quality of the RCT [31]. Communication about the cosmetic appearance of the modified footwear by the physicians involved in a trial can be the key to positively influence compliance [32, 33, 34]. Several participants reported a preference for randomization in the intervention group. Other participants rejected participation altogether, feeling that they had already undergone usual GP care. Offering the deferred footwear intervention if pain symptoms are still present after 6 months follow‐up to participants receiving the control intervention may increase the recruitment rate and enhance adherence. Moreover, the recruitment of incident cases may facilitate the inclusion of individuals who have not yet undergone usual GP care. In addition, as a strong preference for, or against, the intervention or control treatment can limit recruitment [35], it is crucial to provide clear information about both treatments during the recruitment and randomization phase. Utilizing accessible language and explanations in invitation and intervention materials could enhance informing prospective participants. Moreover, the contacting of participants by telephone, mail, and e‐mail requires extra consideration. Effective communication by the research team and involved physicians with (potential) participants should be an integral part of the research process to undermine challenges of an effectiveness trial.

One of the key strengths of our study was the nesting within a quantitative pilot study, which provided unique opportunities for purposive sampling, including both men and women who differed in age, OA symptom duration, and previous experiences, with primary and/or secondary care regarding the first MTP joint OA. Participants were all altruistic and motivated to participate in this qualitative study. However, our findings need to be interpreted in the context of several limitations. First, although all participants provided rich and relevant information, which gave valuable insight into all categories, our sample size of 10 participants was small in relation to commonly recommended thresholds for data saturation [36]. Notwithstanding, this is the first qualitative study within this study population with the aim to identify valuable insights and powerful information that can provide a grounding for designing trials investigating the effectiveness of footwear interventions in this target population. Second, examples of footwear modifications that might be implemented were not consistently shown during each interview. By providing tangible examples, participants may have acquired a more precise comprehension of the modifications, thereby facilitating better feedback and yielding richer data. This approach may have enhanced the reliability and depth of the study findings. Finally, analyses of the interviews indicated that pain symptoms and walking limitations are two significant aspects for the participants. However, it was not clear which important outcome measures were deemed significant by the participants. By asking in the interviews what aspects would benefit from wearing the footwear intervention, we could have clarified this for future studies in the field of firts MTP joint OA. During the course of this study, no established core outcome set for foot OA was available. However, a core outcome set for foot and ankle disorders in rheumatic and musculoskeletal diseases, including foot OA, is currently in development and will help to design future trials [37].

## 5 Conclusion

Our study offers an opportunity to inform the design of a full‐scale RCT on footwear modifications for patients with the first MTP joint OA. Key considerations in the design of a successful future trial includes offering the deferred footwear intervention to the control group, a recruitment strategy focused on recruiting incident cases, and providing clear information about the intervention and control treatment. Moreover, the significance of the cosmetic appearance of modified footwear for the patient population was emphasized.

## Author Contributions

**Carla Braam:** data curation, formal analysis, investigation, project administration, resources, visualization, writing – original draft preparation, writing – reviews and editing. **Sabine Kloprogge:** investigation, visualization, writing – review and editing. **Dieuwke Schiphof:** conceptualization, supervision, writing – review and editing. **Joyce B. J. van Meurs:** conceptualization, writing – review and editing. **Sita M. A. Bierma‐Zeinstra:** conceptualization, writing – review and editing. **Marienke van Middelkoop:** conceptualization, supervision, writing – review and editing.

## Ethics Statement

Ethical approval was obtained from the Medical Ethical Committee of Erasmus Medical Center (MEC‐2022‐0723). All participants provided verbal informed consent.

## Conflicts of Interest

The authors declare no conflicts of interest.

## Supporting information

Supporting Information S1

## Data Availability

Data and material available for this study would require further approval from the corresponding author upon request.
