# Supplementary figures and images for: Patients' Perspectives on Participation in an Effectiveness Study on Footwear Modification for the First Metatarsophalangeal Joint Osteoarthritis: A Qualitative Study
Source: J Foot Ankle Res. 2025 Apr 23;18(2):e70050. doi: 10.1002/jfa2.70050 (PMC12018162; doi:10.1002/jfa2.70050)

**Appendix A: SRQR Checklist**

**
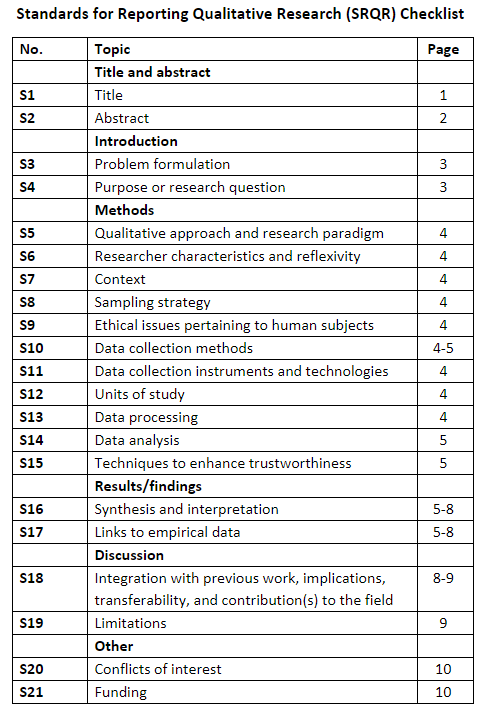
**

**Appendix B: Interview guide**
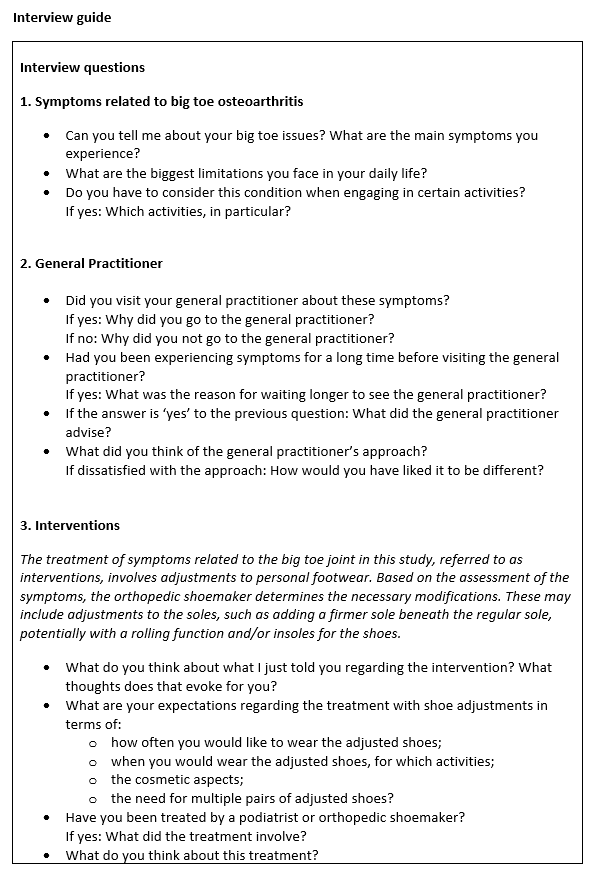


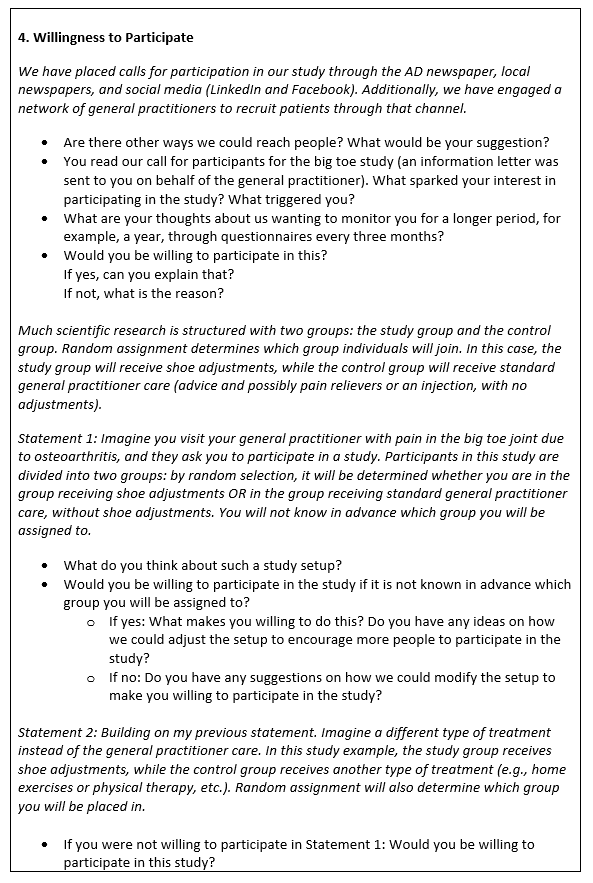


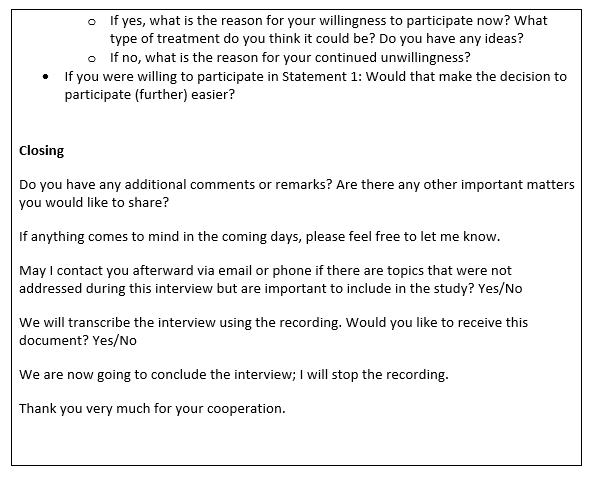

Supplement: Supplementary file 1 — Supporting Information S1 [file JFA2-18-e70050-s001.docx]
